# ScoMorphoFISH: A deep learning enabled toolbox for single‐cell single‐mRNA quantification and correlative (ultra‐)morphometry

**DOI:** 10.1111/jcmm.17392

**Published:** 2022-05-20

**Authors:** Florian Siegerist, Eleonora Hay, Juan Saydou Dikou, Marion Pollheimer, Anja Büscher, Jun Oh, Silvia Ribback, Uwe Zimmermann, Jan Hinrich Bräsen, Olivia Lenoir, Vedran Drenic, Kathrin Eller, Pierre‐Louis Tharaux, Nicole Endlich

**Affiliations:** ^1^ Institute for Anatomy and Cell Biology University Medicine Greifswald Greifswald Germany; ^2^ Section of Human Anatomy Department of Mental and Physical Health and Preventive Medicine University of Campania “Luigi Vanvitelli” Naples Italy; ^3^ 31475 Institute of Pathology Medical University of Graz Graz Austria; ^4^ 39081 Department of Pediatrics II University Hospital Essen Essen Germany; ^5^ Department of Pediatrics University Hamburg‐Eppendorf Hamburg Germany; ^6^ Department of Pathology University Medicine Greifswald Greifswald Germany; ^7^ Department of Urology University Medicine Greifswald Greifswald Germany; ^8^ Nephropathology Institute of Pathology Medical School Hannover Hannover Germany; ^9^ PARCC Paris Cardiovascular Research Centre Inserm Université Paris Cité Paris France; ^10^ NIPOKA GmbH Greifswald Germany; ^11^ 31475 Division of Nephrology Department of Internal Medicine Medical University of Graz Graz Austria

**Keywords:** kidney biopsy, podocyte, renal pathology, SARS‐CoV‐2, super‐resolution microscopy

## Abstract

Increasing the information depth of single kidney biopsies can improve diagnostic precision, personalized medicine and accelerate basic kidney research. Until now, information on mRNA abundance and morphologic analysis has been obtained from different samples, missing out on the spatial context and single‐cell correlation of findings. Herein, we present scoMorphoFISH, a modular toolbox to obtain spatial single‐cell single‐mRNA expression data from routinely generated kidney biopsies. Deep learning was used to virtually dissect tissue sections in tissue compartments and cell types to which single‐cell expression data were assigned. Furthermore, we show correlative and spatial single‐cell expression quantification with super‐resolved podocyte foot process morphometry. In contrast to bulk analysis methods, this approach will help to identify local transcription changes even in less frequent kidney cell types on a spatial single‐cell level with single‐mRNA resolution. Using this method, we demonstrate that ACE2 can be locally upregulated in podocytes upon injury. In a patient suffering from COVID‐19‐associated collapsing FSGS, ACE2 expression levels were correlated with intracellular SARS‐CoV‐2 abundance. As this method performs well with standard formalin‐fixed paraffin‐embedded samples and we provide pretrained deep learning networks embedded in a comprehensive image analysis workflow, this method can be applied immediately in a variety of settings.

## 1 INTRODUCTION

High‐precision analysis of kidney biopsies is key to providing diagnosis and targeted therapies for patients. Recently, several methods have been established to improve the analysis depth of formalin‐fixed paraffin‐embedded (FFPE) kidney biopsies.^1^, ^2^, ^3^ Since the filtration barrier could be morphometrically analysed by 3D‐structured illumination microscopy (3D‐SIM),^3^ the determination of the filtration slit density by PEMP (*podocyte exact morphology measurement procedure*) emerged as a tool that can be combined with co‐staining of multiple proteins.^4^, ^5^ However, antibody‐based quantification of spatial protein abundance has limitations as it depends on individual antibody availability and performance. Additionally, locally secreted factors are typically not captured by immunofluorescence techniques. The use of bulk proteomics and transcriptomics is limited since the expression of frequent cell types like proximal tubule cells can mask transcriptional changes in less frequent cell populations. To circumvent this, tissue has been either manually dissected (e.g. glomeruli from tubulointerstitium),^6^ cell types were enriched by flow cytometry^7^ or single‐cell‐RNA sequencing has been performed.^8^ Unfortunately, the contextual and/or morphological information is lost in all approaches due to mechanical dissociation. As biopsy material is typically limited and interpretation in a spatial context required, correlation of multiple techniques on single sections could increase the degree of information from a single biopsy.

An antibody‐independent way to investigate spatial RNA abundance is in situ hybridization (ISH) which since its first description^9^ has been substantially improved in terms of sensitivity and multiplexing.^10^ Recently, a multitude of methods for single‐mRNA visualization and quantification (smFISH) are available.^11^, ^12^, ^13^ A consistent problem for inter‐sample comparability is that smFISH highly depends on preparation‐dependent RNA integrity. This is due to delays between sampling and fixation, fixation time, quality of the fixative and paraffin‐embedding. To rule out this problem, a stable on‐slide in‐cell reference gene would be required to normalize expression data for different parts of the same biopsy or even over different samples.

To assign transcripts to tissue compartments and individual cell types, reliable identification and segmentation of cellular regions of interest (ROIs) are required. Unfortunately, correlative antibody‐based cell classification is challenging as *smFISH* requires tissue digestion to liberate fixed mRNAs. Additionally, segmentation tasks are typical bottlenecks in image analysis workflows. To overcome this, deep learning (DL) has been used for segmentation and morphometry of kidney biopsies.^14^

Herein, we present scoMorphoFISH (single‐cell correlative Morphometric single‐mRNA FISH), a DL‐accelerated approach for imaging‐based and digital single‐cell single‐mRNA quantification. For the first time, we combined spatial single‐cell expression data with antibody‐based super‐resolved podocyte foot process morphometry. We integrate spatial single‐cell transcriptomic, (ultra‐)morphometric and classic histology over scales as large as whole FFPE sections down to individual foot processes. Early in the SARS‐CoV‐2‐pandemic, it has been noted that besides the lung, the kidney is one of the organs that can be directly infected by the virus.^15^ On the functional side, it has been shown that the degree of albuminuria can predict disease severity in COVID‐19 patients.^16^ In vivo, albuminuria is typically mediated by morphological changes of a single‐cell type, the podocyte. In this cell type, transcriptional changes directly influence cellular morphology, and every change of morphology leads to an impairment of the filtration barrier function. However, until now, no method is available that allows for evaluation of change of transcription, ultrastructural morphometry and classic histology. Herein, we use scoMorphoFISH to evaluate single‐cell mRNA abundance, single‐cell SARS‐CoV‐2 infection and ultramorphometry in a case of COVID‐19‐associated kidney disease.

## 2 MATERIALS AND METHODS

### 2.1 Sample preparation

After immersion fixation in 3% PFA overnight at room temperature, kidneys were embedded in paraffin using standard protocols. Care was taken that the temperature did not exceed 60°C. 5 µm FFPE tissue sections were mounted on superfrost slides and air‐dried at room temperature. Human renal tissue specimens were obtained from the Pathology Department of Hôpital Européen Georges Pompidou, Assistance Publique‐Hôpitaux de Paris, Paris, France. Human tissue was used after obtaining informed consent from all patients, and kidney biopsy collection was approved by the Institut National de la Santé et de la Recherche Médicale Ethics Committee (Institutional Review Board 00003888, approval 13–087; FWA00005831 National Institutes of Health, Office of Human Research Protection) and the local ethics committee (Comité de Protection des Personnes Ile de France IV, Institutional Review Board: 00003835. approval 2015/73NICB). Kidney biopsy specimens were collected in compliance with all relevant ethical regulations, and those with sufficient tissue for immunohistochemical evaluation after the completion of diagnostic workup were included. Similarly, kidney biopsies of the Departments of Pathology of Hannover, Graz and of the Department of Pediatric Nephrology Essen were used. Additionally, we used anonymized excess healthy kidney tissue of tumour nephrectomies from the Department of Urology of the University Medicine Greifswald. Sample transfer adhered to all relevant local and national ethical guidelines. The transfer and use of sections of an anonymized COVID‐19 nephropathy kidney biopsy were approved by the ethics committee of the Medical University of Graz, Austria.

### 2.2 Multiplex fluorescence in situ hybridization and immunofluorescence staining

For multiplex *smFISH*, the ACDbio RNAscope Multiplex Fluorescent V2 Kit per manufacturer's description with following adaptations: For heat‐induced epitope retrieval sections were boiled for 15 min in ACDbio antigen retrieval buffer after deparaffinization in xylene. After protease treatment for 15 min at room temperature, complementary probes were hybridized for 2 h at 40°C in the ACDbio HybEZ oven. Sections were stored in 5× SSC (Sigma) overnight. Hybridized probes were detected by complementary amplification probes after HRP‐binding signals were detected using Opal 520, 570 and 690 dyes diluted 1:1500 in TSA amplification buffer (ACDbio). After the last detection step, sections were collected in 1× PBS, blocked with 1% normal goat serum, 1% foetal bovine serum, 1% bovine serum albumin and 0.5% cold fish gelatin for 45 min at room temperature. While blocking, primary affinity‐purified rabbit anti‐podocin antibodies were mixed with Alexa Fluor 488‐conjugated secondary dual monoclonal recombinant alpaca anti‐rabbit IgG VHH nanobodies (nano secondaries, Chromotek) in blocking solution for a final concentration of 1:150 primary and 1:1000 secondary nanobody. Sections were incubated with the antibody mix at 4°C overnight. For the collapsing FSGS sample, primary NPHS2 antibodies were detected using AlexaFluor 750 secondary antibodies. Slides were washed in three changes of 1× PBS and nuclei counterstained with 0.1 mg/ml DAPI. Slides were rinsed in A.dest and mounted in Mowiol for microscopy (Carl Roth). Until being imaged, sections were stored at 4°C in the dark.

### 2.3 Correlative histology

After being whole‐slide imaged, slides were immersed in 37°C 1× PBS for 1 h. Coverslips were gently removed, and the mounting medium washed out in 3 changes of 1× PBS. After that, routine PAS staining was performed as described before.^3^ Sections were mounted in Eukitt (Carl Roth).

### 2.4 Imaging

To obtain confocal laser scanning micrographs, a Leica TCS‐SP5 system was used. Micrographs were acquired using a 40× 1.2 NA oil immersion objective with a voxel size of 189 × 189 × 500 nm (xyz). For whole‐slide near‐infrared imaging, an Olympus FV3000 system with a 20×, 0.8 NA air objective equipped with a 405, 488, 561 and 640 nm laser lines and an external NIR‐unit with a 730 nm laser line was used. The whole section was imaged as tiled stacks over 2 µm with a voxel size of 222 × 222 × 500 nm. Tile data were stitched within the Olympus FV3000 CellSense software. For super‐resolution 3D‐structured illumination microscopy, a Zeiss Elyra PS.1 system (Carl Zeiss Microsystems) or a Nikon N‐SIM‐E was used as described before.^3^ Whole‐slide images of PAS‐stained sections were acquired on a Leica SCN400 slidescanner. SCN files were imported and processed with QuPath (v0.3.0).

### 2.5 Deep learning

Using the Google Colab‐based ZeroCostDL4Mic notebooks,^17^ we trained a U‐Net,^18^, ^19^ a deep learning‐based neural network. Glomerular tuft outlines determined by NPHS2^+^ glomerular capillaries in stacks of confocal laser scanning micrographs were manually segmented and saved as ROIs in FIJI.^20^ The ROIs were then exported as binary 8‐bit masks in a corresponding image stack in which glomerular area and background were coded as intensity 255 and 0, respectively. Both image stacks were exported as individual corresponding 512 × 512 px tiff files with matching names and uploaded in two separate GoogleDrive‐folders as source and template training files. After training, a separate set of files was used for quality control purposes. The UNet model was trained with 200 epochs on 200 paired image patches (image dimensions: (1024, 1024 px), patch size: (512, 512 px)) and an initial learning rate of 3.0000002e‐36, using UNet 2D ZeroCostDL4Mic. Key python packages used include tensorflow (v 0.1.12), Keras (v 2.3.1), numpy (v 1.19.5) and cuda (v 11.0.221Build cuda_11.0_bu.TC445_37.28845127_0). The training was accelerated using a Tesla K80 GPU. Following parameters were used: number_of_epochs 200; patch_size 512 × 512; batch_size 4; number_of_steps 23; percentage_validation 10; initial_learning_rate 3.0000002e‐36. The readily trained network was exported and saved for subsequent predictions. Predictions were performed in the DeepImageJ plugin by installing the readily trained network. Predicted glomerular regions of interest (ROIs) are saved by the script to the respective source folder and called later by the macro script to define intra‐ and extraglomerular cells and transcripts.

The StarDist^21^ 2D model was trained from scratch for 400 epochs on 4 paired image patches (image dimensions: (2048, 2048 px), patch size: (2048, 2048 px)) with a batch size of 2 and a mae loss function, using the StarDist 2D ZeroCostDL4Mic notebook (v 1.12).^20^ Key python packages used include tensorflow (v 0.1.12), Keras (v 2.3.1), csbdeep (v 0.6.1), numpy (v 1.19.5) and cuda (v 10.1.243). The training was accelerated using a Tesla P100GPU. The dataset was augmented by a factor of 10 using Augmentor.^22^ Following parameters were used: number_of_epochs 400; patch_size 2048 × 2048; batch_size 2; number_of_steps 30.0; percentage_validation 10; n_rays 32; grid_parameter 2; initial_learning_rate 0.0003. The trained StarDist model was imported to Fiji and is automatically called by the scoMorphoFISH script.

### 2.6 Script development

ImageJ macros were developed in the IJ1 macro language in FIJI.^20^ The script requires several different pre‐installed plugins: BioFormats to import C‐LSM data, StarDist which requires the CSBDeep, and StarDist update sites in Fiji, rsFISH,^23^ deepImageJ, and Read and Write Excel. Data in the multichannel tiffs should be ordered with the immunofluorescence channel in C1, *smFISH* in C2/3 and DAPI in C4.

The script can perform different tasks. If glomerular outlines are stained by immunofluorescence:

1. *Glomerular vs*. *tubulointerstitial transcript counter*: For two‐channel smFISH + NPHS2 IF + DAPI. The script asks first for the source folder of the multichannel tiff stacks. The script uses the trained U‐Net to predict the glomerulus segmentation mask from the glomerular staining, takes the outlines of the mask as an ROI, and calculates the intra‐ and extraglomerular area. It then differentially segments intra‐ and extraglomerular cells and counts transcript in the single‐cell ROIs. Output is two‐channel intra‐ and extraglomerular single‐cell transcripts, total transcripts intra‐ and extracellular, intra‐ and extraglomerular area. If no glomerular staining is present, the script will ask if glomerular outlines should be segmented manually. If nuclear IF is present, immunofluorescence positive cells are segmented using the trained StarDist network, and expression is differentially measured IF positive and negative cells. This part is established for podocytes but works with every strong nuclear marker.
2. *Podocyte transcript counter*: For two‐channel smFISH + WT1 IF + DAPI. The script asks first for the source folder of the multichannel tiff stacks and then manually creates the glomerular outline masks. Podocytes are identified by: intraglomerular position, WT1 and DAPI positivity. Podocytes are segmented with the same model as all nuclei. Podocyte morphometric parameters: Total number/glomerular cross‐section, Feret's diameter are exported for estimated glomerular morphometry (podocyte density). Output is intra‐, extraglomerular, total, podocyte single‐cell dual‐channel transcripts.

Output data are exported in one accumulated.xls file per folder analysed. The script works in batch processing mode and processes all tiff files in a directory.

### 2.7 2D density plots

For the creation of 2D‐density plots, the R spatstat package was used: After thresholding and binarization of the smFISH channels, xy‐positions of the transcript spots were detected using RS‐FISH and exported as csv files from ImageJ. XY positions were loaded to R‐studio (version a1.3.1093) and plotted using the density plot function of the SpatStat library.

### 2.8 Statistics

Statistical analysis was performed in Prism 9.3.1 (GraphPad). Normality was checked with a Kolmogorov–Smirnov test. In the case of Gaussian distribution and two compared groups, a Student's *t*‐test with Welch's correction for non‐equal SDs was performed. For more than two groups, a Kruskal–Wallis test with Dunnett's multiple comparison test was performed for non‐parametric data, one‐way ANOVA with Dunnett's multiple comparisons for parametric data. Correlation analysis of co‐expression data was performed with a simple linear regression analysis. The best fit line of the linear regressing including its 95% confidence band is plotted.

## 3 RESULTS

### 3.1 Spatially resolved normalized single‐cell single‐mRNA visualization

**FIGURE 1:**
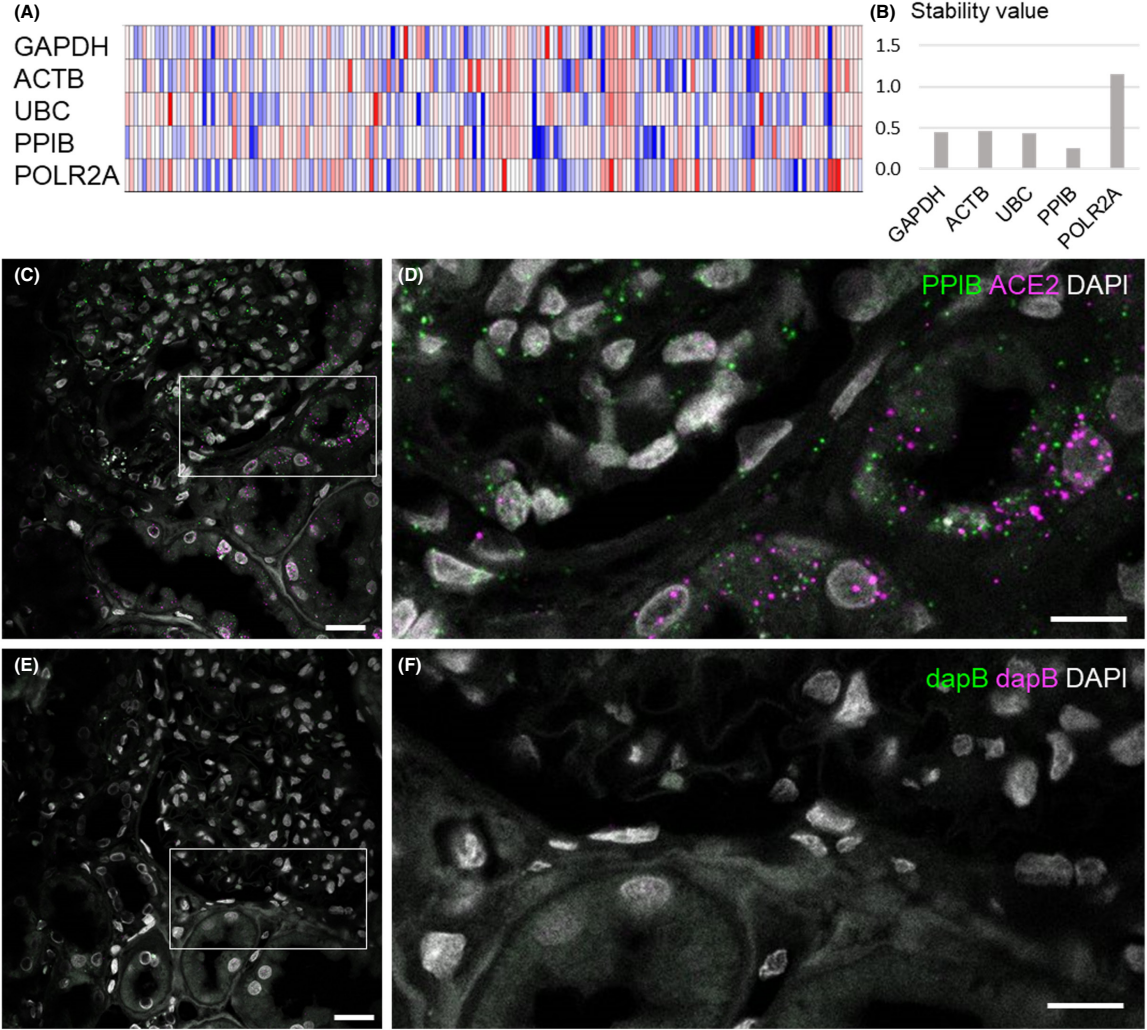
Evaluation of an on‐slide reference gene. The Nephroseq database was screened for the five reference genes GAPDH, ACTB, UBC, PPIB and POLR2A. The heatmap of microarray expression data of the Ju CKD dataset is shown in (A). These data were evaluated in terms of stability over samples using the Normfinder algorithm. With a stability value of 0.257, PPIB was identified as the most stable gene (B). Images in (C and D) show PPIB and ACE2 mRNA dual‐labelling in FFPE human kidney sections. While ACE2 was predominantly expressed in tubular cells, PPIB is abundant in all cells. Single transcript spots can be clearly distinguished. Negative controls with a probe targeting the bacterial dapB gene (E and F) show specificity of the smFISH‐signals. Scale bars represent 40 µm in the overview images and 20 µm in the magnifications

To account for RNA integrity differences in archived formalin‐fixed and paraffin‐embedded (FFPE) material and to normalize mRNA‐expression levels, we wanted to identify an on‐slide reference gene. Such genes should be constantly expressed and not regulated themselves. We screened the Nephroseq database for the reference genes *ACTB*, *GAPDH*, *POLR2A*, *UBC* and *PPIB*. In a glomerular disease microarray dataset (Figure 1A), *PPIB* was the most stable gene (Figure 1B). Performance of *PPIB* as a reference gene was exemplarily demonstrated for a dual *smFISH* together with *ACE2* (Figure 1C,D). *PPIB* was homogeneously and strongly expressed (8.1 ± 4.5 transcripts per cell) (Figure 1C). Negative controls did not show a signal (Figure 1E,F).

### 3.2 Immunofluorescence‐based cell classification and Deep Learning‐enabled tissue segmentation

**FIGURE 2:**
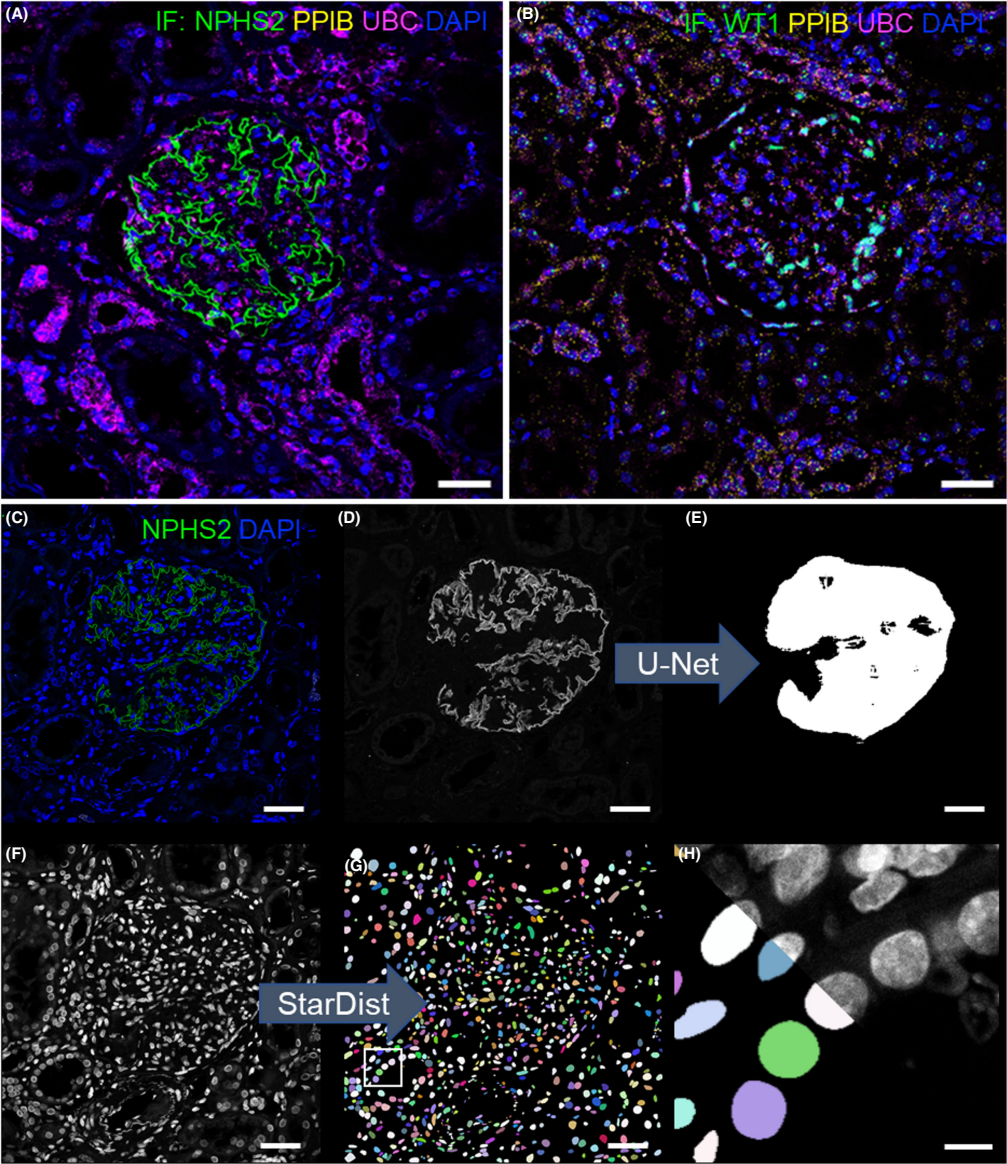
Combination of smFISH and immunofluorescence. The micrograph in (A) shows combined dual‐smFISH with podocin (NPHS2) immunofluorescence. Image (B) exemplarily shows smFISH combined with WT1 tyramide signal amplified‐immunofluorescence. A UNet was trained to segment glomerular outlines of podocin‐stained glomeruli as shown Figure S1A. As shown in (D and E), glomerular outlines were predicted from podocin immunofluorescence micrographs (C) which were used as segmentation masks (E). Panel (F–H) shows how cell nuclei were segmented by the predictions of the trained StarDist network (Figure S1B). The scale bars in a‐g represent 40 and 5 µm in (H)

To assign transcripts to tissue compartments or cell types, we established immunofluorescence protocols that perform with smFISH. Glomeruli were labelled with single‐step anti‐podocin staining (Figure 2A). Less abundant antigens like WT1 were amplified by fluorescent tyramide signal amplification (TSA, Figure 2B).

To establish virtual DL‐segmentation‐based tissue‐microdissection, we custom‐trained the two DL networks UNet^18^, ^19^ and StarDist^21^ with datasets of 200 manually segmented glomeruli and 1033 cell nuclei, respectively (Figure S1). As shown in Figure 2C–H, outlines of glomeruli and cell nuclei of raw images were predicted with high reliability and accuracy by the trained networks. Compared to manually segmented ground truths, the trained networks reached a precision of 90% in the detection of cell nuclei (*n* = 76) and 93% in the detection of glomeruli (see validation data in Figure S2).

### 3.3 scoMorphoFISH: Quantitative analysis of compartment and cell type‐specific mRNA abundance

**FIGURE 3:**
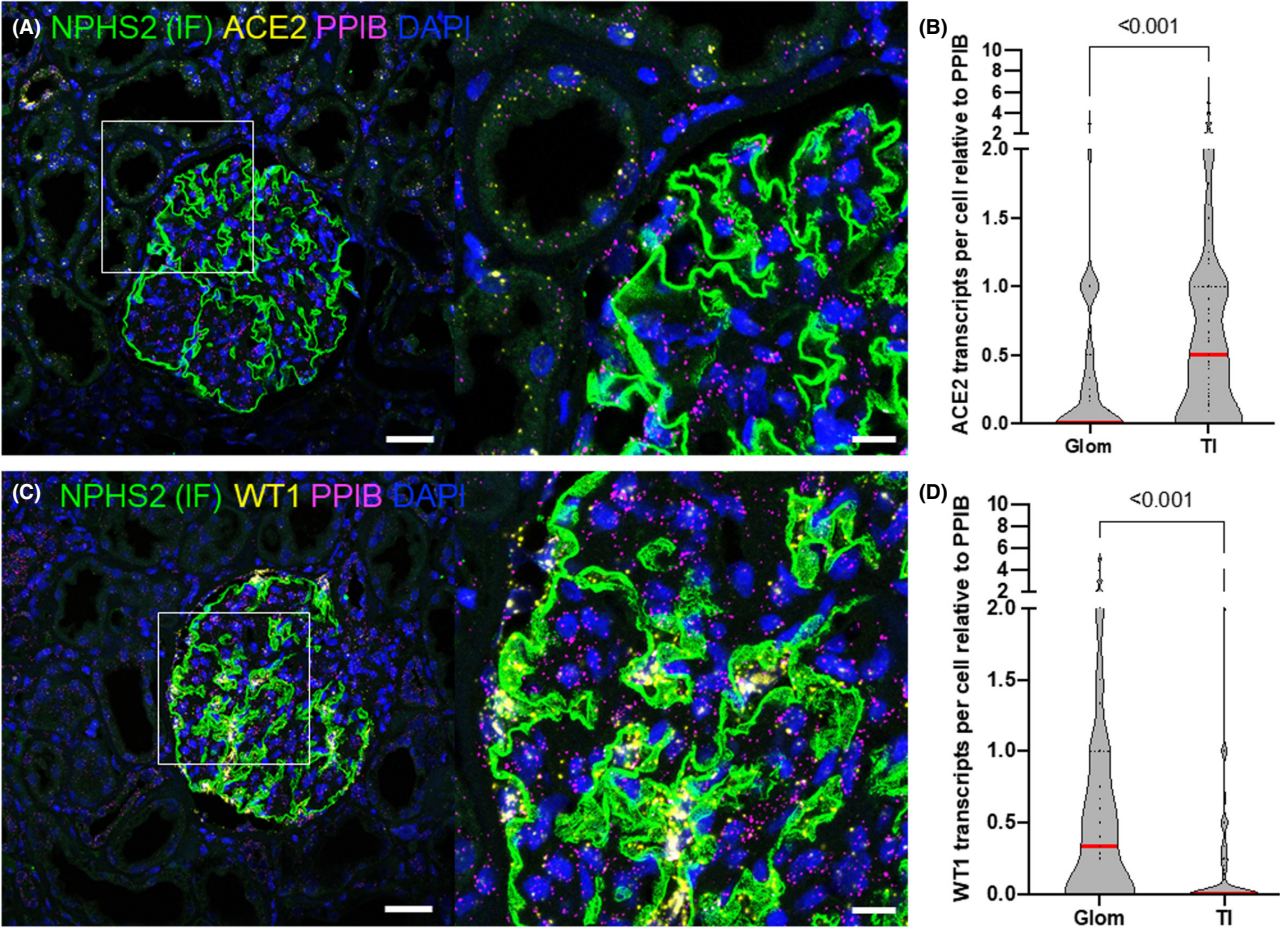
Normalized single‐cell expression of ACE2 and WT1 of glomerular versus extraglomerular cells. Micrographs in a show dual smFISH for ACE2 and PPIB combined with podocin (NPHS2) immunofluorescence. Violin plots in b show statistically significant higher expression of ACE2 in tubulointerstitial (TI) to glomerular (Glom) cells. ACE2: *n* = 329 cells WT1 *n* = 698 cells. *p* > 0.001, two‐way Student's *t*‐test for normal‐distributed data. Scale bars represent 40 µm

To spatially map mRNA transcripts in intact kidney tissue, we applied the RS‐FISH algorithm that uses radial symmetry to approximate transcript localizations.^23^ As demonstrated in Figure S3, RS‐FISH was more precise than the classic thresholding‐based segmentation approach. To automatize normalized *smFISH* transcript annotation to DL‐segmented cells and tissue compartments, we established an open‐source ImageJ script including the trained DL networks and RS‐FISH. As proof‐of‐principle, we quantified the spatial abundance of *ACE2* and *WT1*, which showed *ACE2* clustering in tubulointerstitial cells while *WT1* was highly enriched in the glomerular cell fraction (Figure 3A–D).

**FIGURE 4:**
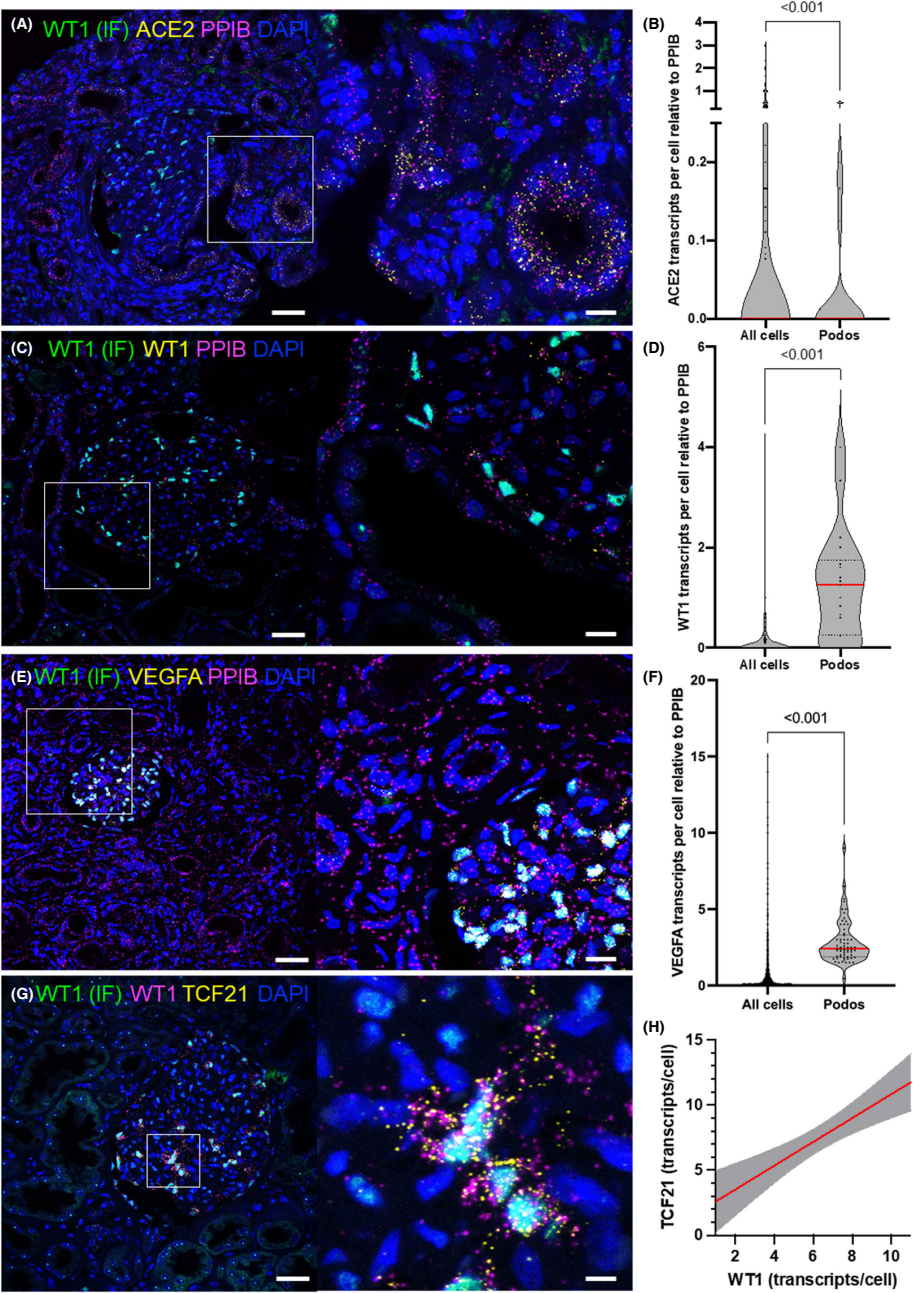
Normalized single‐cell expression and co‐expression analysis. Normalized single‐cell expression of ACE2 (A–B) WT1 (C–D) and VEGFA (E–F) of the podocyte fraction versus all cells. (ACE2: *n* = 324 cells, WT1: *n* = 377, VEGFA: *n* = 1447 cells). Panel G‐H shows single‐cell co‐expression analysis of TCF21 and WT1. The positive correlation in the plot indicates TCF21 and WT1 co‐expression (*R* ^2^ = 0.35, *p* > 0.0001, 95% confidence interval in grey, data from *n* = 40 podocytes of 3 individual glomeruli)

Irrespective of cellular identity, mean *ACE2* expression was 0.251 normalized transcripts/unclassified cell versus 0.07 normalized transcripts/podocyte (Figure 4A,B). Vice versa, *WT1* was ninefold enriched in podocytes in comparison with all cells (Figure 4C,D). Additionally, we quantified the abundance of *VEGFA* mRNA, a secreted factor for which immunostainings are typically not suitable for quantification. Shown in Figure 4E,F is single‐cell expression data of *VEGFA*, which strongly clustered in the podocyte fraction. As shown in Figure 4G,H, a positive correlation of *TCF21* and *WT1* single‐cell expression is demonstrated (*n* = 40 podocytes of three glomeruli, *R* ^2^ = 0.35, *p* > 0.0001).

### 3.4 Correlative super‐resolution podocyte foot process morphometry, multiplex smFISH and standard histology

**FIGURE 5:**
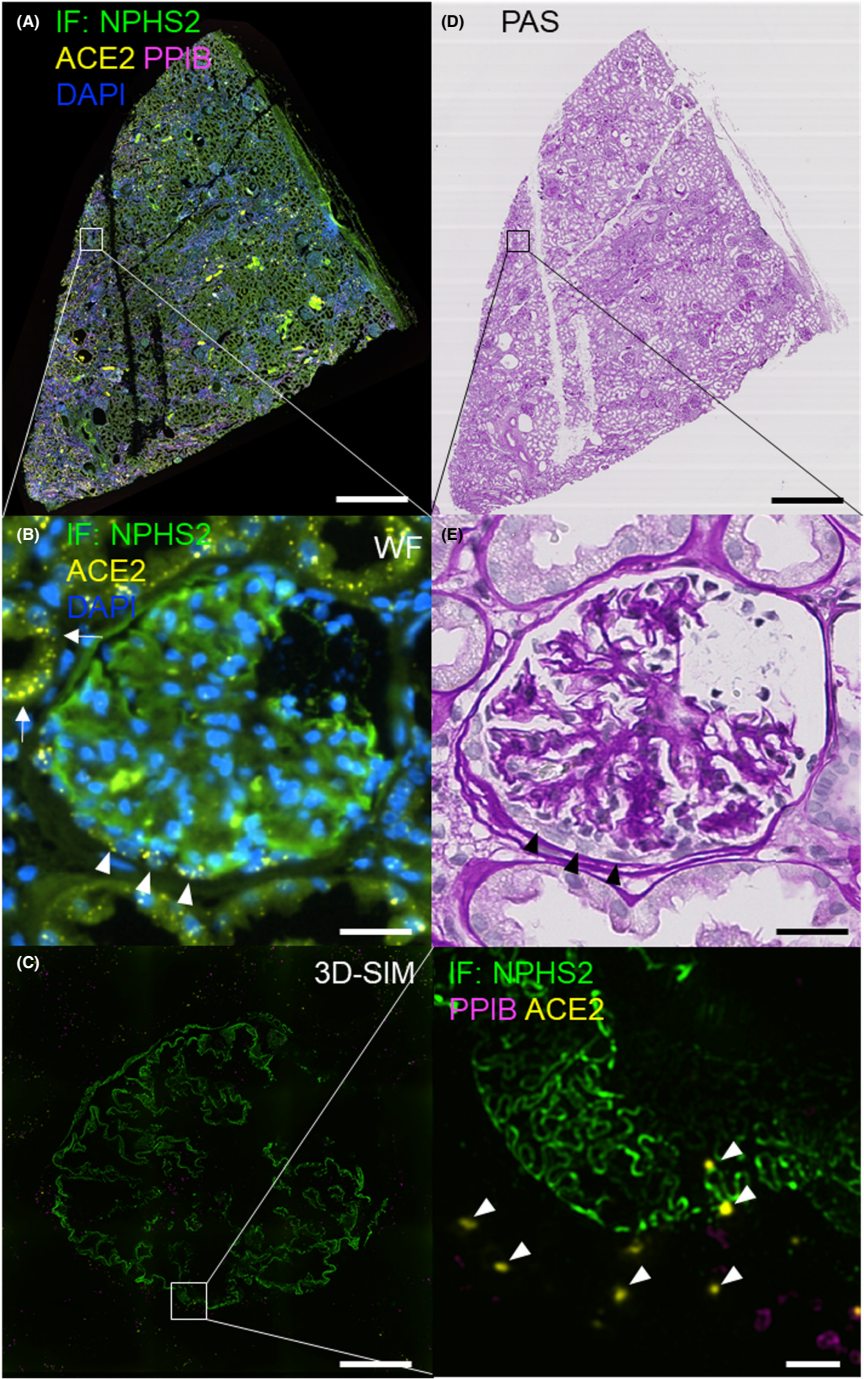
Correlative single‐cell transcript quantification, podocyte ultramorphometry and histology. Subsequent processing of the same FFPE section for scoMorphoFISH and classic histology enables correlative assessment of local mRNA‐expression, podocyte foot process morphology and classic histologic aspects. FFPE sections were whole‐slide scanned and aligned. Micrograph (A) shows a for‐channel fluorescence overview over a whole section. Zoomed in (B) is a single glomerulus in wide field microscopy with ACE2 expression in tubular cells (arrows) and focally in the glomerulus (arrowheads). This area has been resolved with 3D‐SIM (C), visualizing the NPHS2‐positive filtration slit which showed broadened foot processes in areas with ACE2‐expression. These data can be correlated with the histologic aspect in (D and E) that shows parietal basement membrane thickening and cuboidal parietal epithelial cells in this area (arrowheads in (E))

To correlate single‐podocyte mRNA expression with changes of the local filtration slit morphology, we imaged multiplexed *smFISH* and podocin immunofluorescence stainings with 3D‐SIM. For correlation of smFISH and classic histology, sections were retrieved from the mounting medium after imaging, PAS‐stained, and again whole‐slide‐imaged. To efficiently handle whole‐slide data, above‐described algorithms have been adapted to perform DL predictions and transcript quantification these data. As shown in Figure S4A, PAS morphology was sufficient for histopathological assessment, although tissue has been *smFISH*‐processed. As shown in Figure 5A,D, side‐by‐side evaluation of whole‐slide widefield, 3D‐SIM and PAS images allowed parallel evaluation of single‐cell mRNA expression, filtration slit structure and classic morphology. Filtration slits were resolved with a mean resolution of 125 ± 12 nm together (Figure 5C). PEMP filtration slit morphometry did not differ between *smFISH*‐processed and native kidney samples (Figure S4B–D). Local mRNA expression and podocyte ultrastructure can be correlated as exemplified for *ACE2* transcript localization in Figure 5C where yellow dots (arrowheads) correspond to single *ACE2* transcripts. Interestingly, besides tubular *ACE2* expression (arrows in Figure 5B), glomerular *ACE2* expression in this glomerulus was present but restricted to podocytes in an area with cuboidal, likely activated parietal epithelial cells (arrowheads in Figure 5C,E). A second glomerulus of the same section with normal parietal epithelial cells did not show any *ACE2* expression (Figure S5). Remnant podocytes in globally sclerotic glomeruli showed podocyte foot process effacement but preserved *VEGFA* expression (Figure S6).

### 3.5 ACE2 regulation in glomerular disease

**FIGURE 6:**
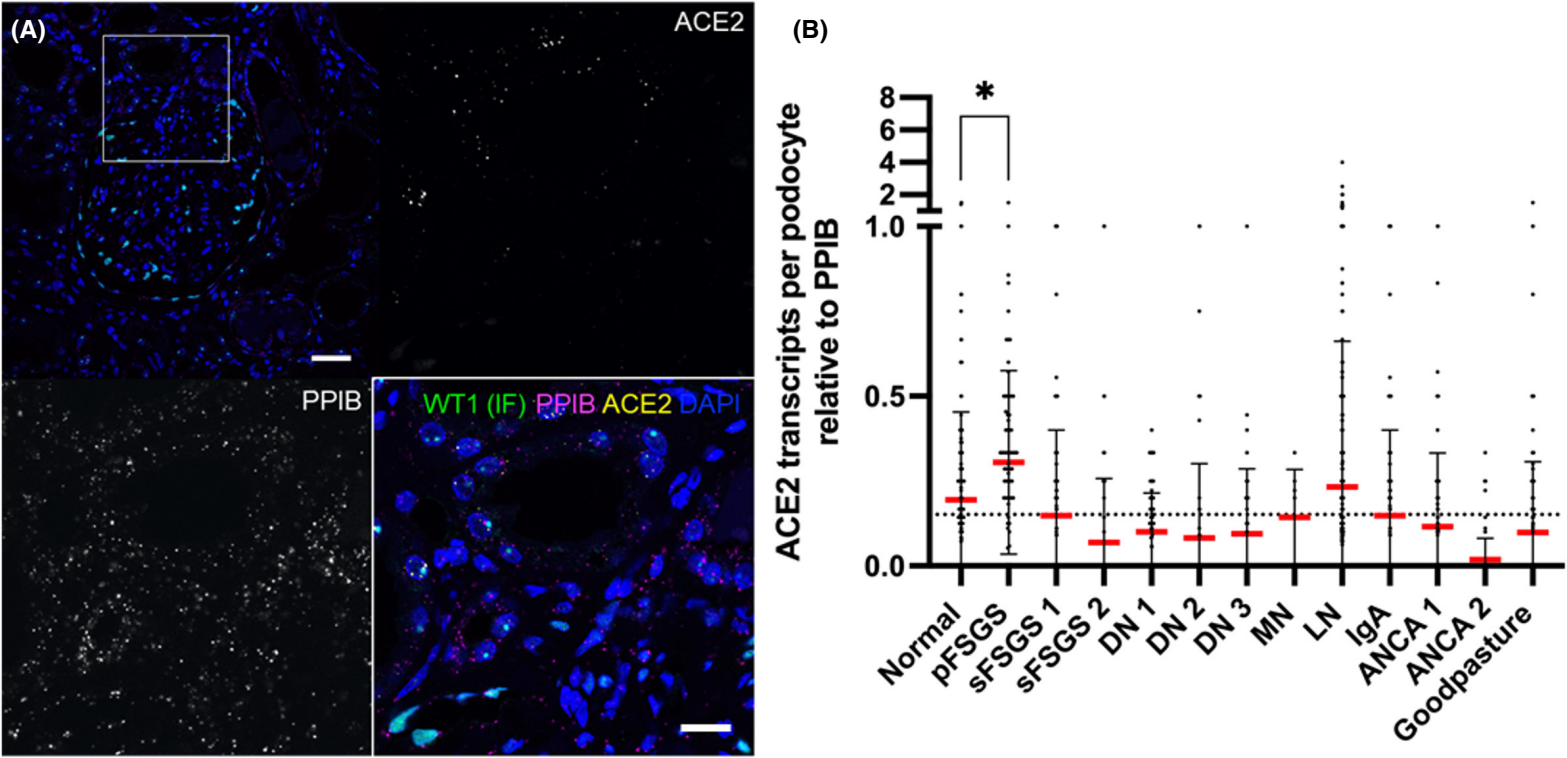
Podocyte ACE2 expression in various biopsies of glomerular diseases. (A) Combined smFISH and WT1 immunofluorescence. The horizontal dashed line in (B) represents the mean expression over all samples. One primary FSGS biopsy shows elevated podocyte‐ACE2 expression (*p* < 0.05 in a Kruskal–Wallis test with Dunn's multiple comparison test for non‐parametric data)

SARS‐CoV‐2 has been found in both tubular cells and podocytes.^15^, ^24^ This is surprising as podocytes express massively lower levels of the respective entry receptor *ACE2* (Figure S7). However, since we have found local podocytic *ACE2*‐upregulation in damaged glomeruli (Figure 5), we investigated its expression in different glomerulopathies. As shown in Figure 6A, we analysed single‐podocyte *ACE2* expression in 13 different biopsies of patients diagnosed for FSGS (primary and secondary), diabetic nephropathy (DN), membranous nephropathy (MN), Lupus nephritis (LN), IgA nephropathy, ANCA‐positive glomerulonephritis and Goodpasture syndrome. While in general, *ACE2* expression was very low, one single primary FSGS biopsy showed statistically significantly elevated podocyte *ACE2* mRNA levels (Figure 6B).

### 3.6 SARS‐CoV‐2 RNA and ACE2 transcripts in a COVID‐19 nephropathy kidney biopsy

Early after the emergence of the COVID‐19 pandemic, a correlation between SAS‐CoV2 infection and collapsing glomerulopathy has been demonstrated.^25^ However, it is still unclear whether this entity is a reaction of a direct viral infection of glomerular cells or a secondary mechanism. While in autopsy material, viral RNA could be demonstrated in a subset of patients,^15^ the visualization of the virus in kidney biopsies of living patients failed in most cases.^24^

**FIGURE 7:**
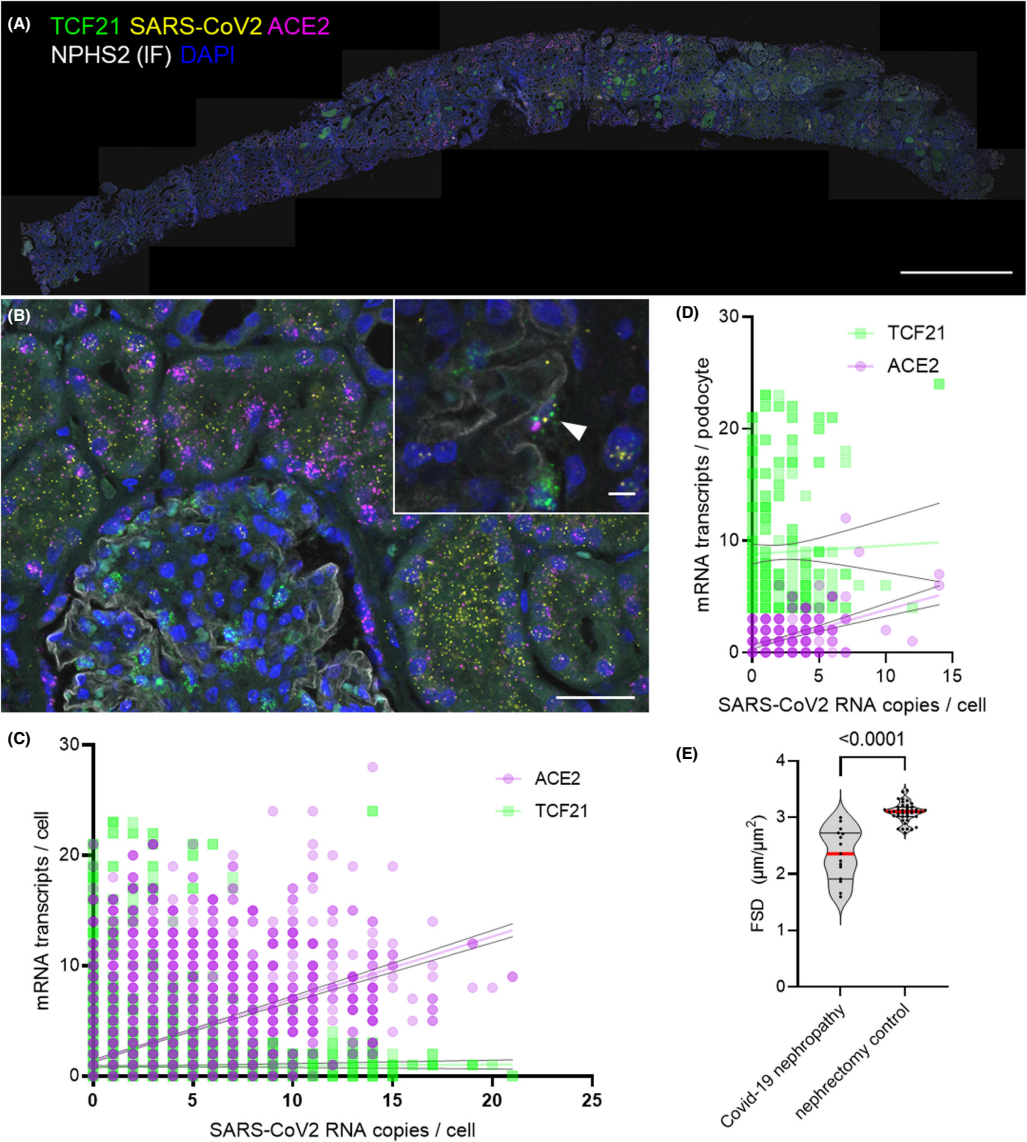
Single‐cell expression analysis of ACE2 and TCF21 mRNA and SARS‐CoV‐2 RNA. A whole‐slide scan of a kidney biopsy diagnosed for collapsing FSGS stained for TCF21 and ACE2 mRNA and SARS‐CoV‐2 RNA together with podocin (NPHS2) immunofluorescence (A). As demonstrated in b, mainly proximal tubular cells showed double‐expression of ACE2 and SARS‐CoV‐2 RNA, but also SARS‐CoV‐2‐infected podocytes could be found (zoom insert in (B)). A positive correlation of single‐cell ACE2 but not TCF21 mRNA expression with intracellular abundance of SARS‐CoV‐2 RNA could be described for unclassified cells (C) and also for podocytes selected by TCF21 expression and intraglomerular position (D). The filtration slit density quantified by PEMP was statistically significantly decreased as compared with beforehand published values in healthy nephrectomy tissue (E).^3^ Scale bars represent 1 mm in A, 25 µm in B and 5 µm in the zoom insert

We applied the above‐described method on FFPE sections of kidney biopsy of a patient diagnosed with COVID‐19‐associated collapsing FSGS. We visualized SARS‐CoV‐2 RNA together with *ACE2* mRNA and *TCF21* mRNA to detect podocytes combined with anti‐podocin immunofluorescence. All negative controls for the SARS‐CoV‐2 probe (SARS‐CoV‐2 probe on healthy pre‐COVID‐19 sections and a probe targeting the bacterial gene dapB) did not show a signal (Figure S8). A lot of SARS‐CoV‐2 RNA could be located in the tubular compartment with a focus on proximal tubular cells (Figure 7A,B). Interestingly, viral RNA was not disseminated over the kidney tissue but rather restricted to single nephrons. In the nephrons affected, triple‐positive (*TCF21*, *ACE2 and SARS*‐*CoV*‐*2*) podocytes could be found (Figure 7B, arrowhead).

Using scoMorphoFISH, a total of 5394 cells of this single section were segmented and single‐cell expression data for *TCF21*, *ACE2* and *SARS*‐*CoV*‐*2* was quantified. Over all cells, intracellular *SARS*‐*CoV*‐*2* abundance was positively correlated with *ACE2* expression (simple linear regression, *p* < 0.0001, *R* ^2^ = 0.2) while this correlation was not found for *TCF21* (*p* = 0.4384, Figure 7C).

From this dataset, podocytes were selected by *TCF21* expression and intraglomerular position (DL‐segmentation of NPHS2 immunofluorescence). Also, in this selective subset of cells, *ACE2* single‐cell expression levels were positively correlated with intracellular abundance of *SARS*‐*CoV*‐*2* RNA (*p* < 0.0001, *R* ^2^ = 0.23, *n* = 300 cells) while no correlation between *TCF21* and *SARS*‐*CoV*‐*2* was found (*p* = 0.6154, Figure 7D).

PEMP analysis demonstrated a statistically significant lower filtration slit density in comparison with healthy control tissue originating from tumour nephrectomies indicating significant podocyte foot process effacement (Figure 7E). The high variance of the FSD is a strong indicator for a rather focal than global foot process effacement.

## 4 DISCUSSION

Herein, we present *scoMorphoFISH*, a straightforward approach for automated digital spatial *in situ* mRNA quantification, Deep Learning‐accelerated antibody‐based tissue segmentation combined with correlative podocyte foot process morphometry (PEMP) in routine kidney biopsies. We used *smFISH* to in situ‐visualize single mRNA transcripts inside intact FFPE kidney tissue. Besides *smFISH*, other techniques are used to investigate spatial mRNA abundance: Spatial transcriptomics with local reverse transcription and subsequent RNA sequencing provides a large number of transcripts per area. Unfortunately, it lacks performance with routinely generated clinical samples and is inferior to *smFISH* in terms of sensitivity and spatial resolution.^26^ The most significant limitation for the performance of *smFISH* is the initial preparation quality which can vary significantly among different centres. To overcome this, we have established *PPIB* as an on‐slide reference gene to normalize target transcript expression levels across samples or even in different parts of the same sample. We used material derived from five different origins (hereof four centres that contributed archived samples). Sections from one centre posed a suboptimal RNA integrity, which could be due too short, too long fixation or too high embedding temperature which can be detrimental for in‐tissue RNA integrity. We deliberately established *scoMorphoFISH* on rather challenging archived clinical samples with a higher degree of heterogeneity compared to standardized material generated in a basic‐research context. Thus, the functionality of this method is not species‐limited and readily usable in rodent models for which we have already shown the performance of PEMP.^4^ Due to high sensitivity and specificity, this technique poses excellent functionality in routinely generated and archived formalin‐fixed paraffin‐embedded (FFPE) material with the potential to correlate spatial transcription and morphometric data with corresponding clinical data and disease history. Therefore, *scoMorphoFISH* enables hypothesis testing in sample repositories with archived medical records.

Although intense tissue digestion was required for *smFISH*, super‐resolved podocyte filtration slit morphology was largely unaffected and filtration slit density was in line with previously published values.^3^ Even though we developed *scoMorphoFISH* focusing on glomerular diseases, it can be instantly applied to other tissue compartments and even other organs. Ideally, an antibody can be used to segment respective tissue compartments, which chances we significantly improved when using tyramide signal amplification. In cases in which such antibody is not available, antibody‐independent *smFISH* expression levels can be used to identify respective cell types as we have shown with *TCF21* or *VEGFA* for podocytes.

Typically, tissue segmentation tasks are demanding in heterogeneous sample sets (like kidney biopsies) and therefore traditional bottlenecks in image analysis workflows. DL has great potential to accelerate segmentation tasks and has already been applied to classify glomerulosclerosis^27^ or immunofluorescence‐based glomerular morphometry.^14^ We custom‐trained two DL networks for the virtual microdissection of glomeruli, all cells by DAPI‐fluorescence, and podocytes using nuclear IF‐markers. The barriers to establishing this method in other settings are low as only commercially available reagents are used, high‐quality imaging systems are widely available, data analysis is performed in the open‐source image analysis platform Fiji and the source code and pretrained DL networks are fully available.

Further, we found that although not expressed under healthy baseline conditions, we show that the SARS‐CoV‐2 target *ACE2* can be locally upregulated in damaged podocytes. As collapsing FSGS, a disease entity frequently associated with viral infections was also described in COVID‐19,^28^ we apply the developed workflow to investigate the localization of SARS‐CoV‐2 RNA in a kidney biopsy of a patient suffering of a likely COVID‐19‐associated collapsing FSGS. Herein, we found that the main SARS‐CoV‐2 target were ACE2‐positive proximal tubule cells. Additionally, to tubular cells, a subset of podocytes was SARS‐CoV‐2 positive indicating direct infection of these cells. In this subset of cells, a positive correlation of SARS‐CoV‐2 and ACE2 mRNA could be found. This is not only confirmative of previous work demonstrating that SARS‐CoV‐2 has a tropism for the kidney, but also a first hint that podocyte‐ACE2 upregulation could be a prerequisite for SARS‐CoV‐2 infection of podocytes.^15^, ^24^

As shown here, correlative assessment of spatial in situ single‐cell single‐mRNA abundance and local podocyte ultramorphology is now possible for the first time on scales as large as entire FFPE sections. We believe that scoMorphoFISH is a valuable addition to the kidney tissue analysis toolbox, can aid researchers in hypothesis testing and could be a possible step towards the high‐precision evaluation of kidney biopsies in clinical settings.

## AUTHOR CONTRIBUTIONS

**Florian Siegerist:** Conceptualization (equal); Formal analysis (equal); Funding acquisition (supporting); Investigation (lead); Methodology (lead); Software (lead); Writing – original draft (lead). **Eleonora Hay:** Investigation (supporting); Methodology (supporting); Visualization (supporting); Writing – original draft (supporting); Writing – review & editing (equal). **Juan Saydou Dikou:** Formal analysis (supporting); Investigation (supporting); Methodology (supporting); Software (supporting); Writing – review & editing (supporting). **Marion Pollheimer:** Resources (equal); Writing – review & editing (supporting). **Anja Büscher:** Resources (equal); Writing – review & editing (supporting). **Jun Oh:** Conceptualization (supporting); Resources (equal); Writing – review & editing (supporting). **Silvia Ribback:** Resources (equal); Writing – review & editing (supporting). **Uwe Zimmermann:** Resources (equal); Writing – review & editing (supporting). **Jan Hinrich Bräsen:** Resources (equal); Writing – review & editing (supporting). **Olivia Lenoir:** Resources (equal); Writing – review & editing (supporting). **Vedran Drenic:** Investigation (supporting); Visualization (supporting); Writing – review & editing (supporting). **Kathrin Eller:** Resources (equal); Writing – review & editing (supporting). **Pierre‐Louis Tharaux:** Resources (equal); Writing – review & editing (equal). **Nicole Endlich:** Conceptualization (equal); Funding acquisition (lead); Project administration (equal); Supervision (lead); Writing – review & editing (equal).

## CONFLICTS OF INTEREST

NE serves as CEO and holds shares of NIPOKA GmbH. FS holds shares and VD is employee of NIPOKA GmbH, a startup commercializing PEMP. All other authors report no competing interests.

## Supporting information

Fig S1‐S8

## Data Availability

All source codes of scripts used in this manuscript as well as example data are available online: http://www.github.com/Siegerist
