# Supplementary material for: ScoMorphoFISH: A deep learning enabled toolbox for single‐cell single‐mRNA quantification and correlative (ultra‐)morphometry
Source: J Cell Mol Med. 2022 May 20;26(12):3513–26. doi: 10.1111/jcmm.17392 (PMC9189342; doi:10.1111/jcmm.17392)
Supplement: Supplementary file 1 — Fig S1‐S8 [file JCMM-26-3513-s001.pdf]

ScoMorphoFISH: A Deep-Learning enabled toolbox for single-cell single-mRNA quantification and correlative (ultra-)morphometry

Florian Siegerist[1], Eleonora Hay[1,2], Juan Saydou Dikou[1], Marion Pollheimer[3], Anja Büscher[4], Jun Oh[5], Silvia Ribback6, Uwe Zimmermann[7], Jan Hinrich Bräsen[8], Olivia Lenoir[9], Vedran Drenic[10], Kathrin Eller[11], Pierre-Louis Tharaux[9], Nicole Endlich[1]*

[1]Institute for Anatomy and Cell Biology, University Medicine Greifswald, Greifswald, Germany
[2]Department of Mental and Physical Health and Preventive Medicine, Section of Human Anatomy, University of Campania "Luigi Vanvitelli", Naples, Italy
[3]Institute of Pathology, Medical University of Graz, Graz, Austria
[4]Department of Pediatrics II, University Hospital Essen, Essen, Germany
[5]Department of Pediatrics, University Hamburg-Eppendorf, Hamburg, Germany
[6]Department of Pathology, University Medicine Greifswald, Greifswald, Germany
[7]Department of Urology, University Medicine Greifswald, Greifswald, Germany
[8]Nephropathology, Institute of Pathology, Medical School Hannover, Hannover, Germany
[9]Université de Paris, INSERM, U970, PARCC, F-75015 Paris, France
[10]NIPOKA GmbH, Greifswald, Germany
[11]Division of Nephrology, Department of Internal Medicine, Medical University of Graz, Graz, Austria

Keywords: kidney biopsy, podocyte, SARS-CoV-2, renal pathology, super resolution microscopy

*Address for correspondence:
Prof. Dr. rer. nat. Nicole Endlich
Institute for Anatomy and Cell Biology
University Medicine Greifswald
17487 Greifswald
Germany

Supplementary Figures:

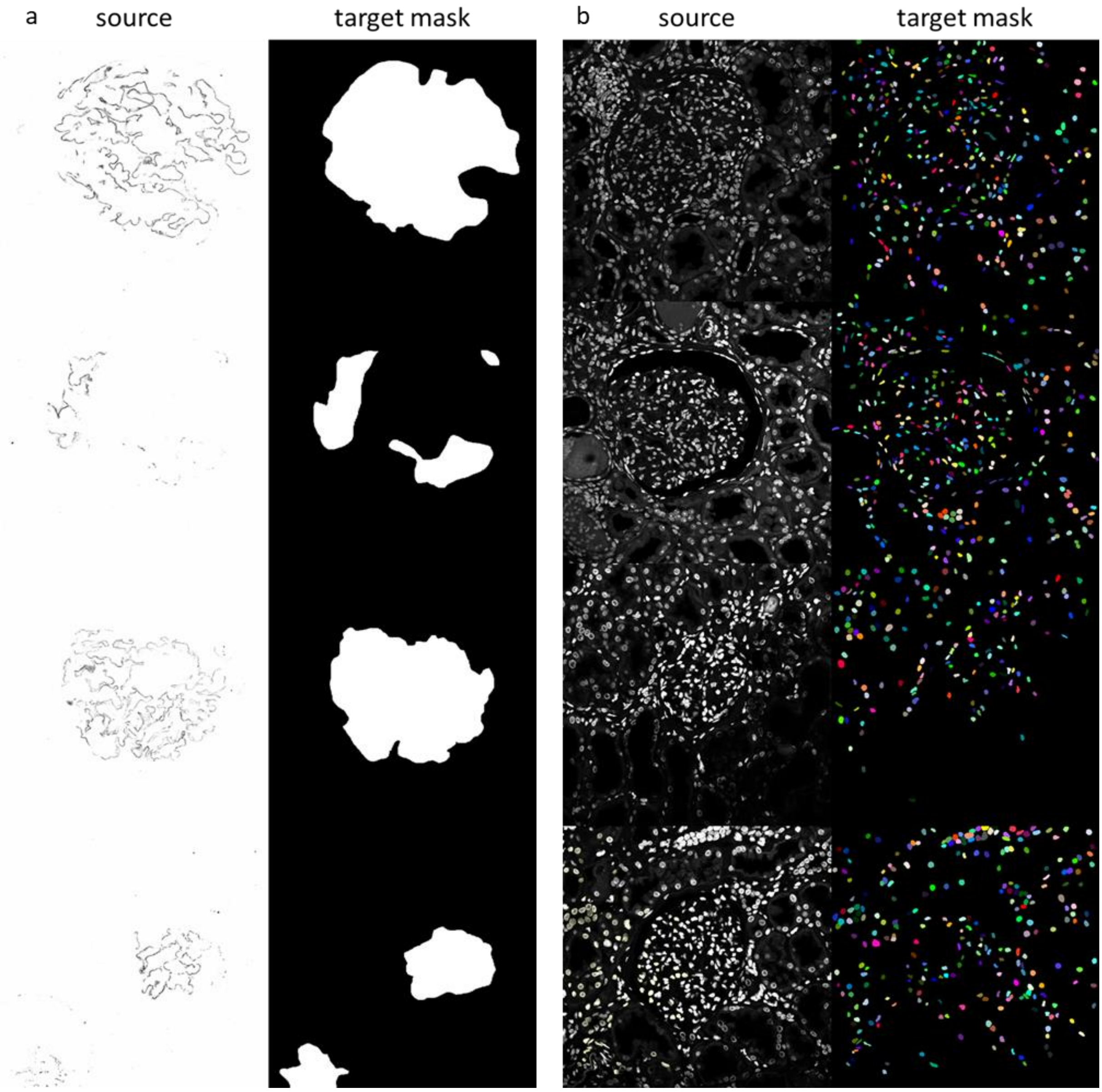

Supplemental Figure 1 Deep learning training datasets for the segmentation of glomeruli in NPHS2-stained kidney section using a UNet (a) or the segmentation of DAPI-labelled cell nuclei (b).

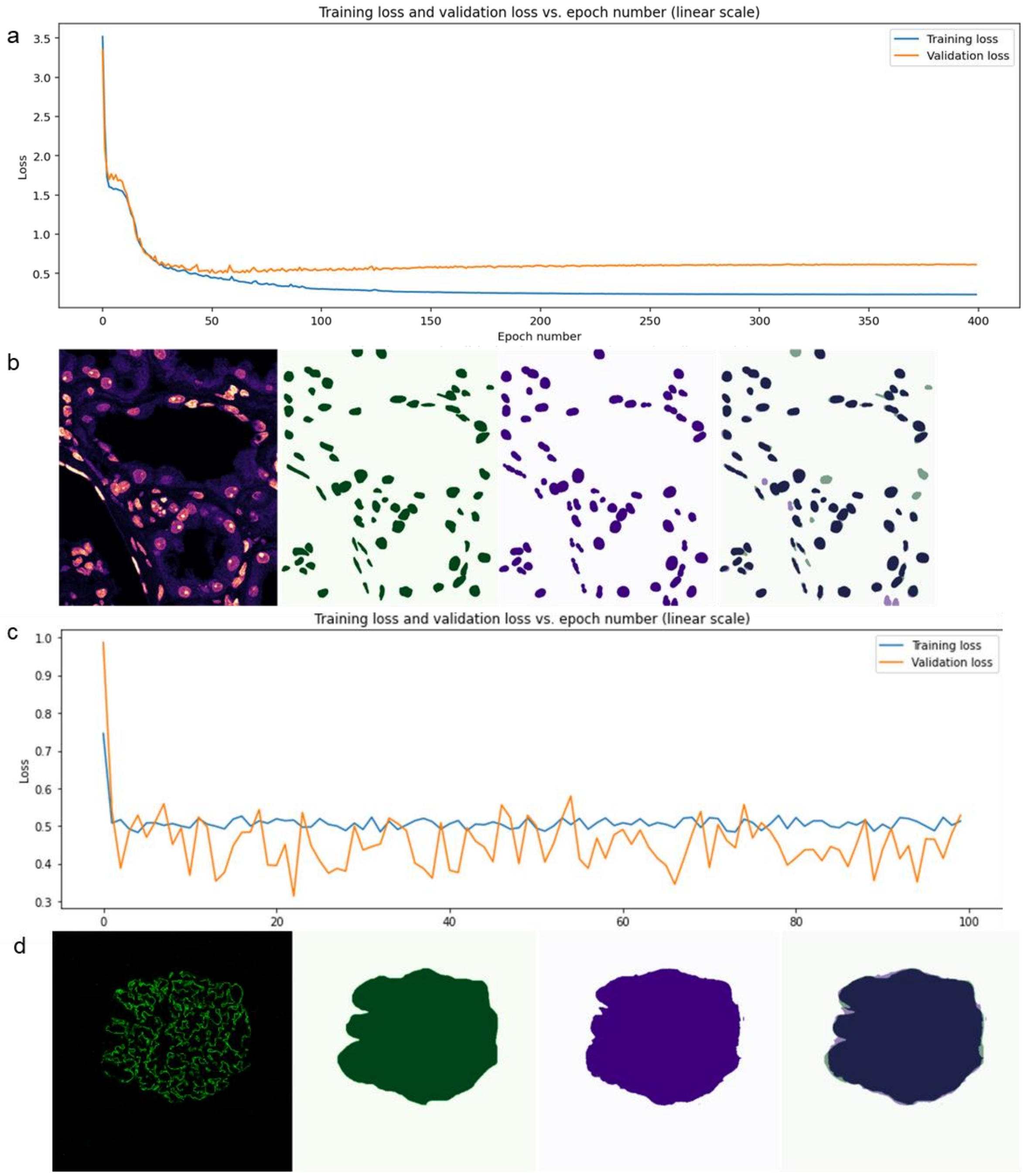

e

Parameter	Value
False positive	7
True positive	66
False negative	9
Precision	0.90411
Recall	0.88
Accuracy	0.804878

Supplemental Figure 2: Deep learning validation data for the StarDist network (a, b) and the UNet c-e). Training and validation loss over the training steps are plotted in a and c. In b and e, comparison of ground-truth (green) and the output of the DL networks (purple) are shown. Quality control data for the StarDist network is shown in e.

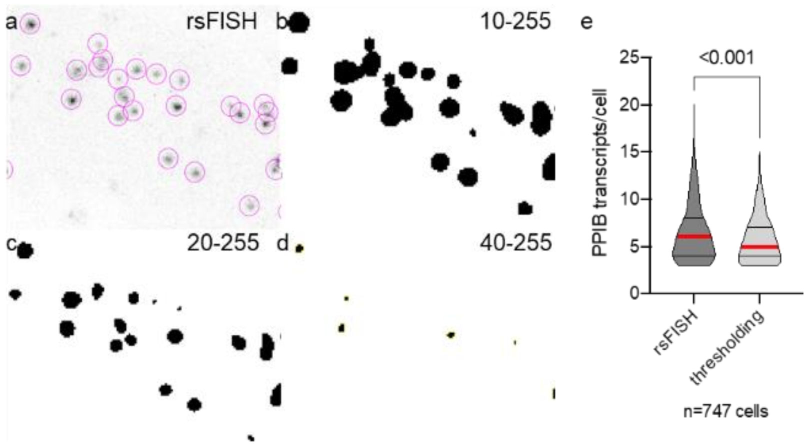

Supplemental Figure 3: RS-FISH detection of smFISH spots. In comparison to classic thresholding, rsFISH localizes more transcripts/cell with higher accuracy. Image a shows the input data together with encircled smFISH transcript localizations detected by the RS-FISH algorithm. In contrast to that, b-d show transcript segmentations after conventional thresholding which either lead to confluent transcript spots (b, c) or lose positive signals (d).

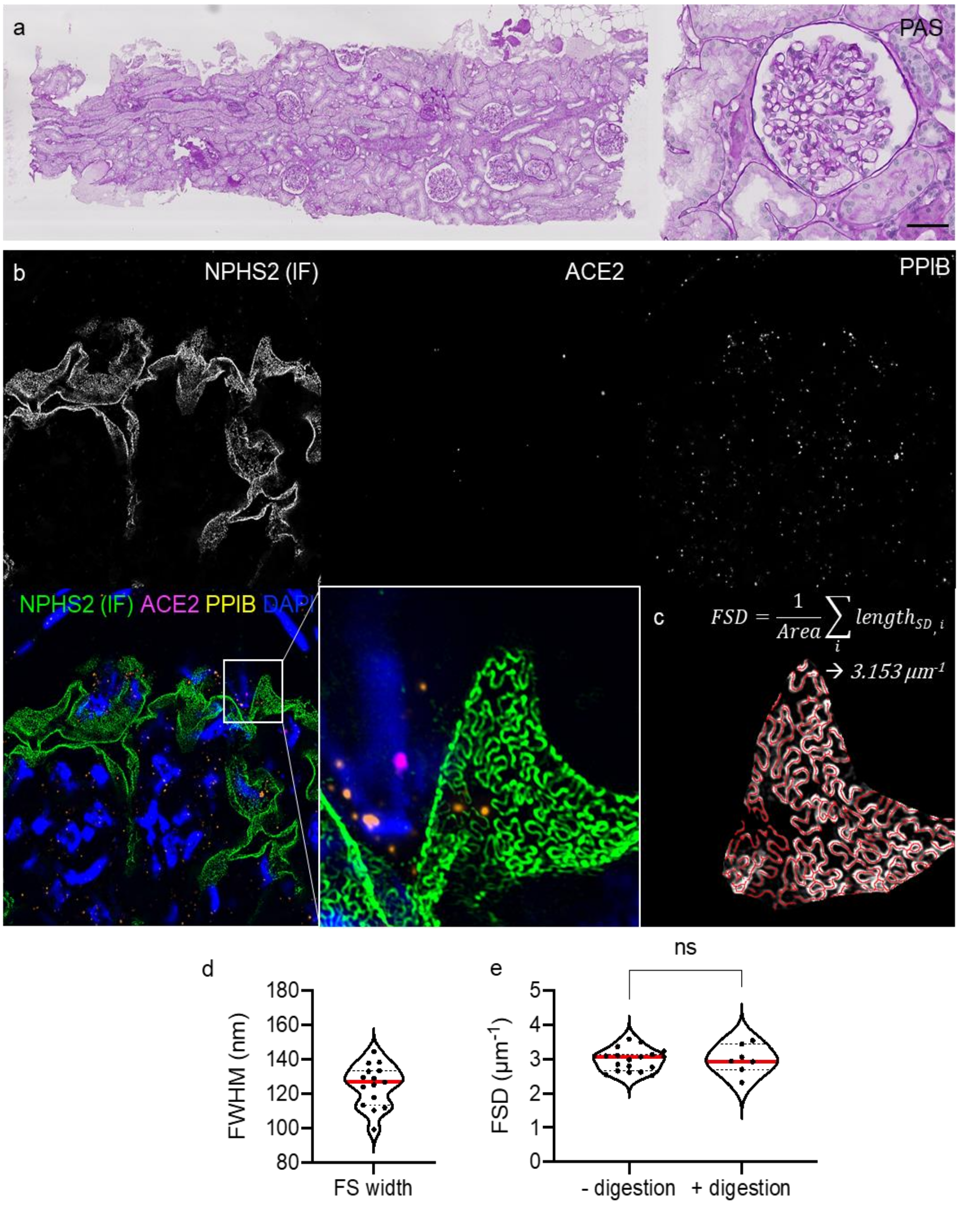

Supplemental Figure 4 Classic PAS histology after tissue digestion showed tissue integrity sufficient for pathohistological assessment (a). Podocyte filtration slits were labelled with a primary-conjugated podocin (NPHS2) antibody (b). Shown in b is the correlative imaging of local podocyte ultrastructure and associated smFISH transcripts As shown in b, filtration slit density was quantified as the total length of the filtration slit per glomerular capillary area. Optical resolution as determined as the full width at half maximum of the sub-diffraction filtration slit was 125 nm±12 nm, sufficient to resolve individual foot processes (d). Digestion required for smFISH had no influence of filtration slit density (e).

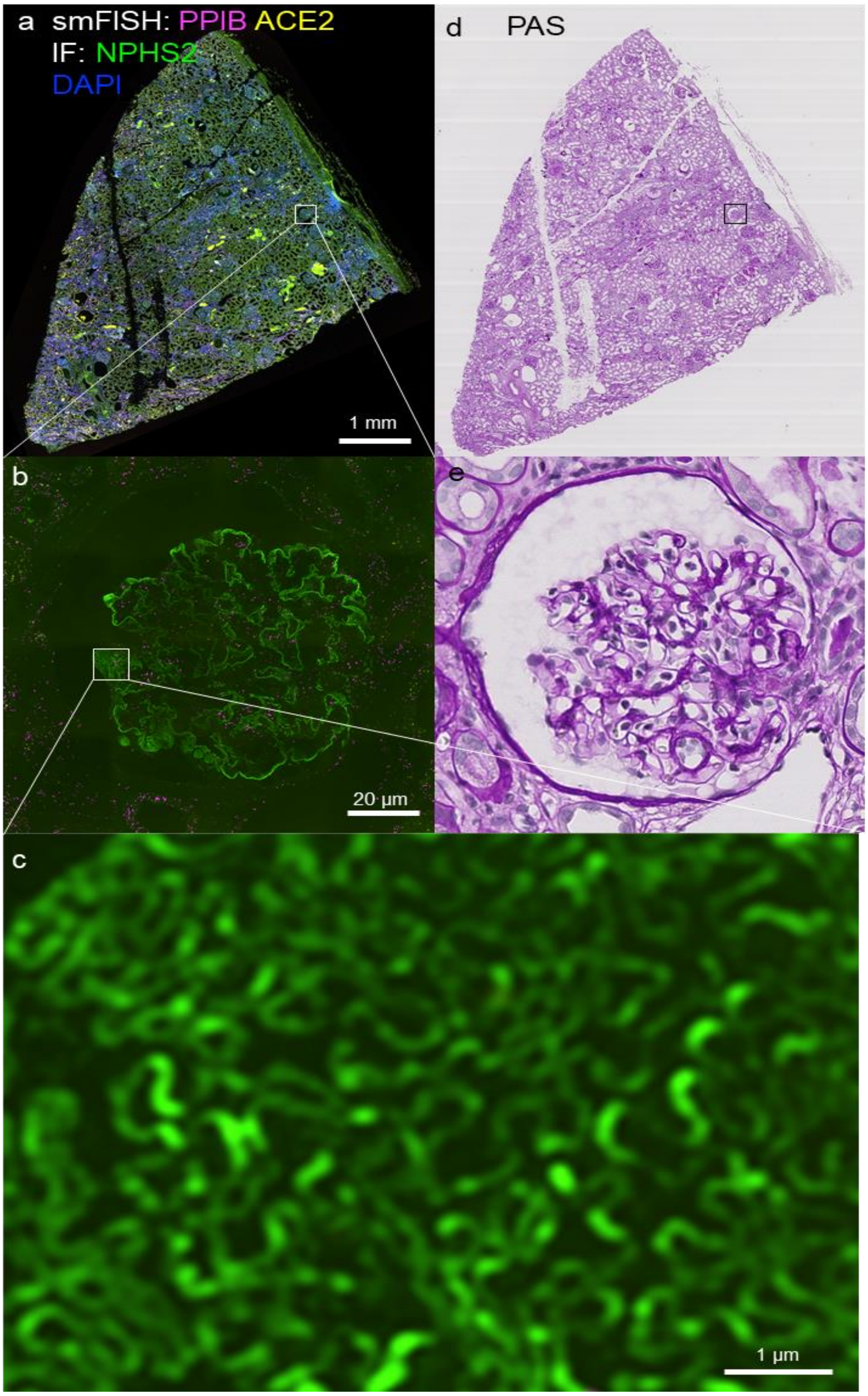

Supplemental Figure 5: Correlative single-cell transcript quantification, podocyte ultramorphometry and histology. A second normalized smFISH and correlative NPHS2-stained (a) glomerulus from main Fig.7. with normal podocyte structure (b,c) and no parietal cell changes (d, e).

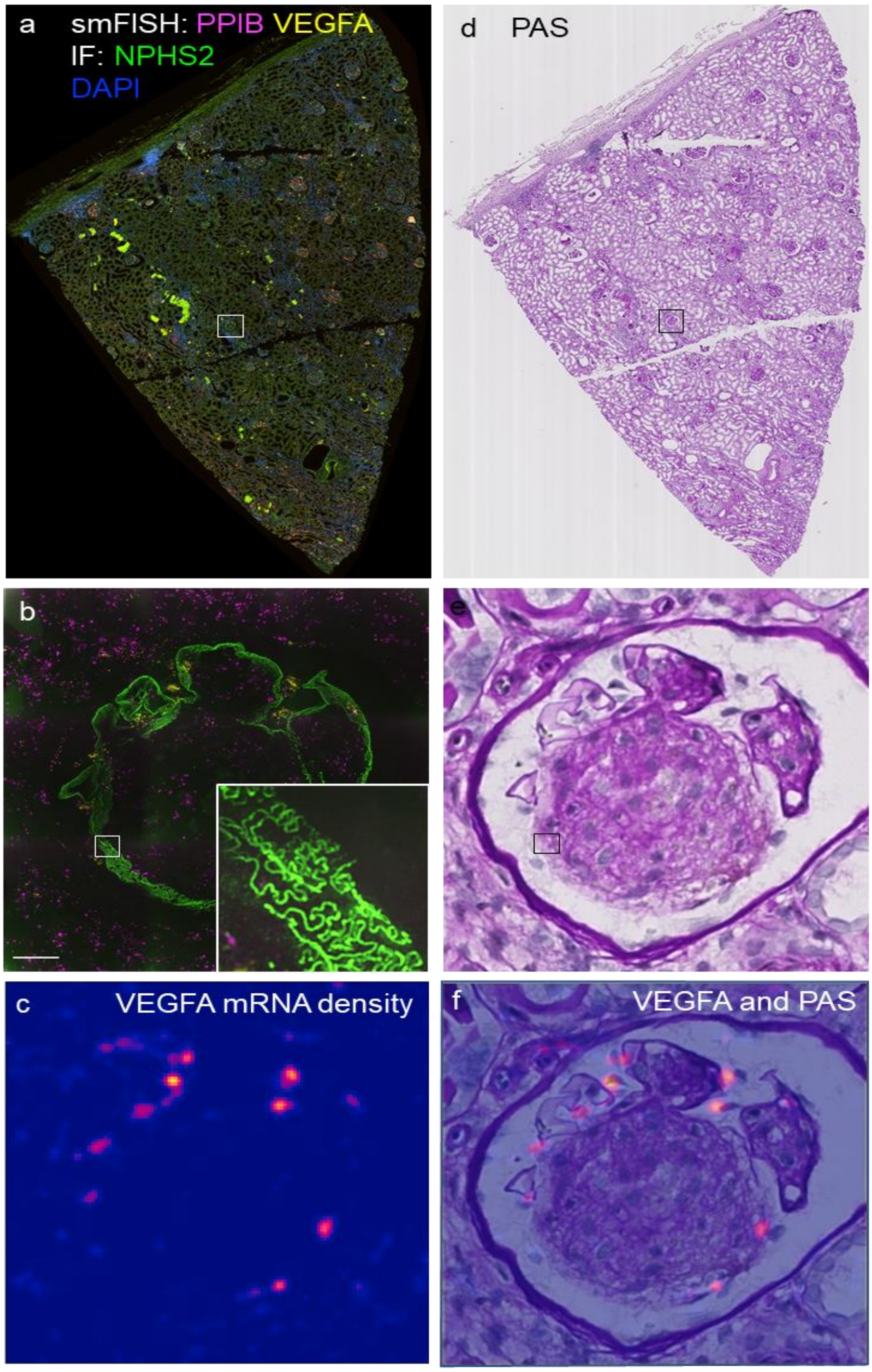

Supplemental Figure 6: Correlative smFISH for VEGFA and PPIB together with super-resolved NPHS2 IF (a). Micrograph and insert in b show central NPHS2-negativity and lateral foot process effacement. Still, VEGFA-expressing cells were present as shown in the VEGFA mRNA density-plots in c. Correlative PAS histology reveals almost global sclerosis (d, e). Local mRNA density can be directly correlated with PAS histology as demonstrated for VEGFA in f. The scale bar in b represents 20 µm.

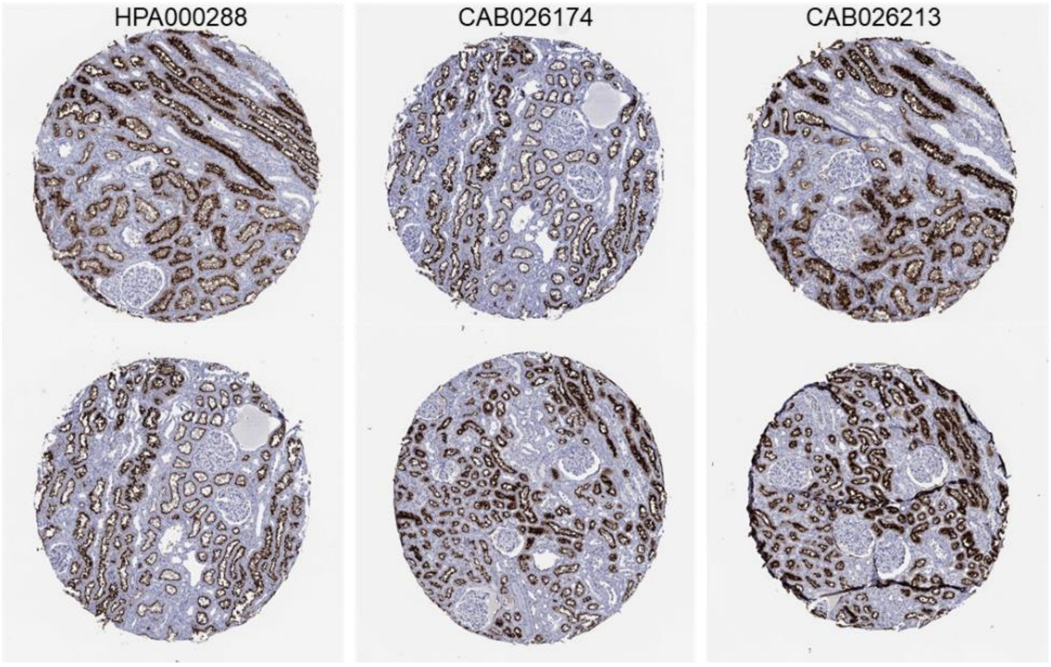

Supplemental Figure 7 ***ACE2 immunohistochemistry with three different antibodies of the Proteinatlas database (www.proteinatlas.org).*** *ACE2 is predominantly expressed in tubular cells.*

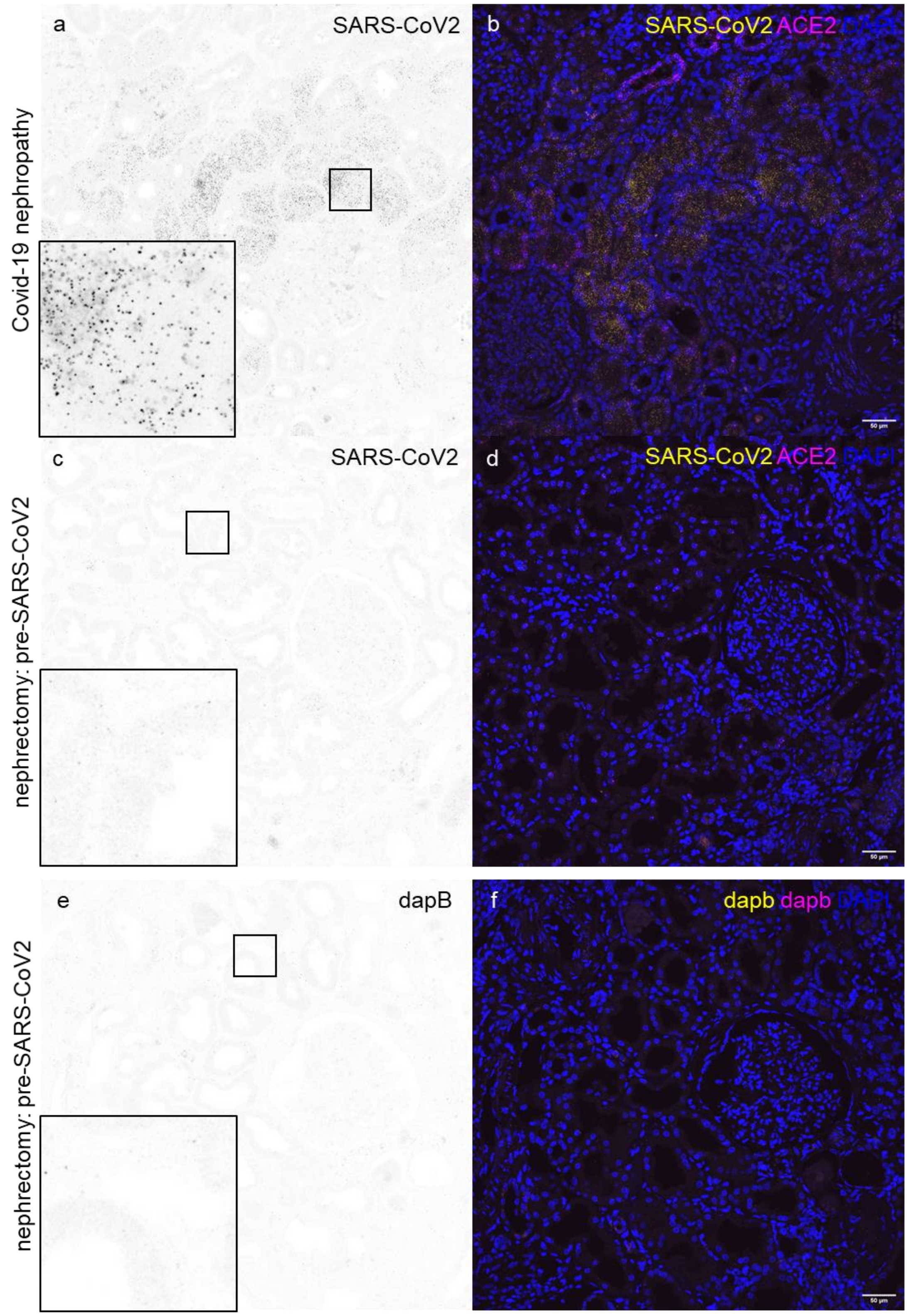

Supplemental Figure 8: Negative controls for SARS-CoV2 smFISH. Anti-SARS-CoV2 smFISH on a Covid-19 associated collapsing FSGS sample shows a strong and positive signal (a, b) while no significant signal could be found in pre-Covid-19 tissue (c, d). The negative control targeting a bacterial gene did not produce fluorescent spots (e, f). The scale bars represent 50 µm
